# Tryptophan degradation in women with breast cancer: a pilot study

**DOI:** 10.1186/1756-0500-4-156

**Published:** 2011-05-26

**Authors:** Debra E Lyon, Jeanne M Walter, Angela R Starkweather, Christine M Schubert, Nancy L McCain

**Affiliations:** 1 Department of Family and Community Health Nursing, Virginia Commonwealth University School of Nursing, 1100 East Leigh Street, Richmond, Virginia, 23298, USA; 2 Department of Adult Health and Nursing Systems, Virginia Commonwealth University School of Nursing, 1100 East Leigh Street, Richmond, Virginia, 23298, USA; 3 Department of Mathematics and Statistics, Air Force Institute of Technology, 2950 Hobson Way, Wright-Patterson Air Force Base, Ohio, 45433, USA

## Abstract

**Background:**

Altered tryptophan metabolism and indoleamine 2,3-dioxygenase activity are linked to cancer development and progression. In addition, these biological factors have been associated with the development and severity of neuropsychiatric syndromes, including major depressive disorder. However, this biological mechanism associated with both poor disease outcomes and adverse neuropsychiatric symptoms has received little attention in women with breast cancer. Therefore, a pilot study was undertaken to compare levels of tryptophan and other proteins involved in tryptophan degradation in women with breast cancer to women without cancer, and secondarily, to examine levels in women with breast caner over the course of chemotherapy.

**Findings:**

Blood samples were collected from women with a recent diagnosis of breast cancer (*n *= 33) before their first cycle of chemotherapy and after their last cycle of chemotherapy. The comparison group (*n *= 24) provided a blood sample prior to breast biopsy. Plasma concentrations of tryptophan, kynurenine, and tyrosine were determined. The kynurenine to tryptophan ratio (KYN/TRP) was used to estimate indoleamine 2,3-dioxygenase activity. On average, the women with breast cancer had lower levels of tryptophan, elevated levels of kynurenine and tyrosine and an increased KYN/TRP ratio compared to women without breast cancer. There was a statistically significant difference between the two groups in the KYN/TRP ratio (*p *= 0.036), which remained elevated in women with breast cancer throughout the treatment trajectory.

**Conclusions:**

The findings of this pilot study suggest that increased tryptophan degradation may occur in women with early-stage breast cancer. Given the multifactorial consequences of increased tryptophan degradation in cancer outcomes and neuropsychiatric symptom manifestation, this biological mechanism deserves broader attention in women with breast cancer.

## Background

Altered tryptophan metabolism and indoleamine 2,3-dioxygenase (IDO) activity are linked to cancer development and progression [1-4]. In addition, these biological factors have been associated with the development and severity of neuropsychiatric syndromes, including major depressive disorder [5-8]. However, this biological mechanism associated with both poor disease outcomes and adverse neuropsychiatric symptoms has received little attention in women with breast cancer (BCA) although women with breast cancer have increased rates of neuropsychiatric symptoms, not only during the acute treatment phase but for some, into survivorship [9-12]

Indoleamine (2,3)-dioxygenase (IDO) is an extrahepatic enzyme that is overexpressed in many cancers and is activated in cancer cells as a strategy for immune escape, a critical aspect of cancer progression [13]. IDO catalyzes the initial and rate-limiting step of the catabolism of tryptophan, an essential amino acid [14]. In cancer, tumors generate an immunosuppressive microenvironment that protects the tumor from host immunity, promotes tumor growth, and attenuates immunotherapeutic efficacy [15]. Tryptophan depletion via IDO is part of the cytostatic and antiproliferative activity mediated by interferon-γ in cells [16,17].

In addition to contributing to an environment that supports tumor development and progression, the biological mechanisms associated with increased TRP degradation may contribute to neuropsychiatric symptoms in persons with cancer through two metabolic pathways. First, inflammatory activation or expression of IDO by tumor cells can lead to elevated levels of IDO activity, which, in turn, increases the degradation of TRP into kynurenine (KYN) leaving less TRP available for other metabolic processes [18]. As TRP is the primary amino acid precursor of serotonin, systemic TRP depletion results in decreased serotonin and melatonin synthesis [19]. In addition, the production and build-up of kynurenic acid, a metabolite of TRP degradation, inhibits the release of glutamate and dopamine [20], important neurotransmitters for regulating neurocognitive function.

The concentration of TRP can also influence levels of tyrosine (TYR), an important amino acid that is converted to levodopa by the enzyme tyrosine hydroxylase (TH) by dopaminergic cells in the brain [21]. In the adrenal medulla, tyrosine is converted into catecholamine hormones, norepinephrine and epinephrine. In addition, the thyroid hormones, triiodothyronine (T3) and thyroxine (T4) in the colloid of the thyroid, are derived from tyrosine and PN symptoms can develop from excessive or deficient levels of T3 and T4 [22]. Therefore, a deficit in TRP availability may alter several key hormones and neurotransmitters involved in maintaining emotional and physical homeostasis.

Secondly, breakdown of TRP through the KYN pathway leads to the production of a series of potentially neurotoxic metabolites, such as quinolinic acid [20]. An elevated level of quinolinic acid can lead to neurocognitive dysfunction as well as other somatic and affective symptoms. Elevated levels of IDO with a subsequent acceleration of TRP degradation along with changes in the availability of key hormones, neurotransmitters, and the accumulation of neurotoxic metabolites could represent an important cascade of events that occur between the proinflammatory state produced by cancer or its treatment and the manifestation of PN symptoms [23-25].

Studies in cancer provide some support for the role of increased TRP degradation in systemic immune activation, severity of physical symptoms, regulation of immune cells within the tumor microenvironment, and in the progression of disease. An increased KYN/TRP ratio and reduced level of TRP correlated with immune activation and reduced quality of life in patients with colorectal liver metastases [26]. In this study, serum TRP was an independent predictor of physical symptom scores measured by the Rotterdam Symptom Checklist and Sickness Impact Profile. In patients with colorectal cancer, the level of IDO expression within tumors correlates inversely with the number of tumor infiltrating cells and the clinical outcome of colorectal cancer patients [27]. Tumor cells with high expression of IDO avoid immune attack by local TRP depletion and the production of proapoptotic TRP catabolites. IDO activity (KYN/TRP ratio) was significantly higher in 123 patients with lung cancer compared to a control (no cancer) group (47.1 ± 21.3 vs. 32.9 ± 9.10, respectively; p < 0.01) [28]. In addition, patients with advanced lung cancer had significantly higher IDO activity and lower TRP concentrations than those in the early stages (p = 0.005 and p = 0.021 respectively) suggesting that TRP degradation may occur more significantly with advanced stage cancer [28]. Increased synthesis of IDO protein was positively associated with impaired survival in women with ovarian serous adenocarcinomas [29]. While these studies suggest critical relationships among TRP metabolism, immune regulation and disease progression in cancer, there has been little study TRP degradation in patients with BCA. Therefore, given the importance of further understanding the biological mechanisms associated with BCA and neuropsychiatric symptoms, a pilot study was undertaken to compare levels of TRP and other proteins involved in TRP degradation in women with BCA to women without cancer, and secondarily, to examine levels in women with BCA over the course of chemotherapy.

## Methods

### Study Design

This was a prospective pilot study to compare levels of TRP and other proteins involved in TRP degradation in women with BCA and women without cancer, and secondarily, to examine how levels change over the course of chemotherapy. Based on past research [9-12], women with BCA typically experience worsening symptoms as treatment progresses, with the most severe neuropsychiatric symptoms occurring after the completion of chemotherapy. Therefore, data collection time points were selected for participants with BCA before the initial dose of chemotherapy was administered and after the cessation of chemotherapy.

### Sample and Setting

For this study, two groups of women were recruited; women recently diagnosed with BCA and a comparison group of women without BCA (confirmed by a negative breast biopsy). Women older than 21 years of age with a first-time diagnosis of Stage I-III BCA and women undergoing breast biopsy for a suspicious breast lesion were invited to participate. Exclusion criteria included previous diagnosis of BCA or any other type of cancer, current use of antidepressants or other medications that interfere with TRP metabolism, and English proficiency.

Participants were recruited from two university health systems in the mid-Atlantic region of the United States. Women with BCA (N = 33) were approached about study participation after their 4^th ^week post-surgery (lumpectomy or mastectomy) but before receiving their first dose of chemotherapy. Participants in the comparison group (N = 24) were approached prior to breast biopsy for a suspicious breast lesion and all participants in this group had a negative biopsy result. Due to the exploratory nature of this study, the sample size estimation was based on having 12 participants for each of the main variables, TRP and IDO. Recruitment continued until there were at least 24 participants in each group. Each participant verbalized understanding and gave informed consent to the respective protocols, which were approved by the university's institutional review board.

### Procedures

Blood samples were obtained using a standard phlebotomy protocol at the time of consent. Blood was collected in a serum separator vacutainer without anticoagulant and allowed to coagulate for 20 to 30 minutes at room temperature. Sera were separated by centrifugation, and all specimens were aliquoted immediately, frozen and stored at -70°C until batch processing [30].

The concentrations of TRP, KYN, and TYR were measured simultaneously by high performance liquid chromatography (HPLC) equipment with fluorescence detection according to Costa et al. [21]. The laboratory of Dr. George Anderson at Yale University performed the assays using previously validated modifications of published methods [31,32]. Samples were prepared by deproteinization after addition of internal standard (α-methyltryptophan). Supernates were directly injected onto a reverse phase HPLC system, and measurements were detected fluorometrically (285 and 345 nm excitation and emission wavelengths, respectively). A quality assessment ("QC") sample was included in all runs and compounds were detected with within-day and day-to-day coefficients of variation (CVs) of less than 5% and 7%, respectively. Using the formula described by Myint et al. [19], IDO activity was estimated as the ratio of KYN to TRP.

### Data analysis

Data analysis was conducted using SAS version 9.1.3 (SAS Institute, North Carolina), and the significance level was set at α < .05. Participants with missing data were not included in the analysis. Means and confidence intervals were used to examine the distribution of the data. Failing equality of variance tests, differences between groups, women with BCA and women without cancer (comparison group), were tested with independent t-tests assuming unequal variances and with independent t-tests assuming equal variances for TRP, KYN, and TYR. Bonferroni corrections for multiple t-tests were employed as necessary.

## Results

The women with BCA and those in the comparison group were similarly aged (means 50.0 ± 11.0 and 47.8 ± 7.6 years respectively, *p *= 0.76). Descriptive statistics for women with BCA prior to and after chemotherapy (baseline and time 2) and women in the comparison group are listed in Table 1.

**Table 1:** Descriptive Statistics for Women in the Comparison Group and Women with Breast Cancer at Baseline (Time 1) and After Completion of Chemotherapy (Time 2)

	n	mean	median	std	stderr	95% LCL	95% UCL
**Comparison Group (Benign Breast Biopsy)**							

Kynurenine (μmol/l)	24	1.98	1.92	0.43	0.09	1.79	2.16

Tryptophan (μmol/l)	24	52.89	51.90	10.44	2.13	48.48	57.30

Kyn/Trp ratio (μmol/l μmol)	24	3.81	3.81	0.88	0.18	3.44	4.18

Tyrosine (μmol/l)	24	81.70	76.63	26.20	5.35	70.64	92.77

Age (years)	24	49.96	48.50	10.98	2.24	45.32	54.60

**Breast Cancer Group**							

**Time 1 (Baseline - Prior to Induction of Chemotherapy)**							

Kynurenine (μmol/l)	33	2.27	2.05	1.13	0.20	1.87	2.67

Tryptophan (μmol/l)	33	48.86	47.62	10.68	1.86	45.08	52.65

Kyn/Trp ratio (μmol/l μmol)	33	4.62	4.10	1.88	0.33	3.95	5.28

Tyrosine (μmol/l)	32	85.43	81.49	19.12	3.38	78.53	92.32

Age (years)	33	47.76	50.00	7.58	1.32	45.07	50.45

**Time 2 (After Completion of Chemotherapy)**							

Kynurenine (μmol/l)	30	2.00	1.97	0.66	0.12	1.75	2.24

Tryptophan (μmol/l)	30	48.14	49.82	10.81	1.97	44.11	52.18

Kyn/Trp ratio (μmol/l μmol)	30	4.23	3.99	1.36	0.25	3.72	4.74

Tyrosine (μmol/l)	30	86.44	83.99	20.76	3.79	78.69	94.19

**Figure 1:**
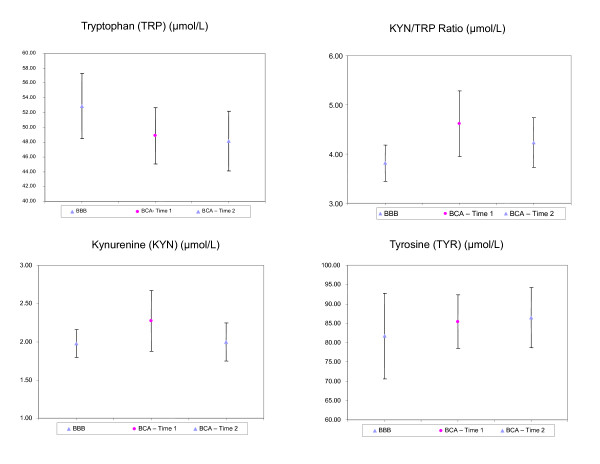
**Means and 95% CIs of Biological Factors for Women in the Comparison Group and Women with Breast Cancer at Baseline (Time 1) and After Completion of Chemotherapy (Time 2)**.

On average, the women with BCA had higher levels of KYN, TYR, and IDO activity (KYN/TRP ratio) and lower levels of TRP than women in the comparison group. Means and 95% confidence intervals for these proteins are plotted in Figure 1. The KYN pathway is constitutively active in the liver, and a basal concentration of KYN is typically found in serum at approximately 1.92 ± 0.58 μmol/L in healthy individuals, whereas concentrations of free TRP range from 40 to 100 μmol/L [31].

**Figure 2:**
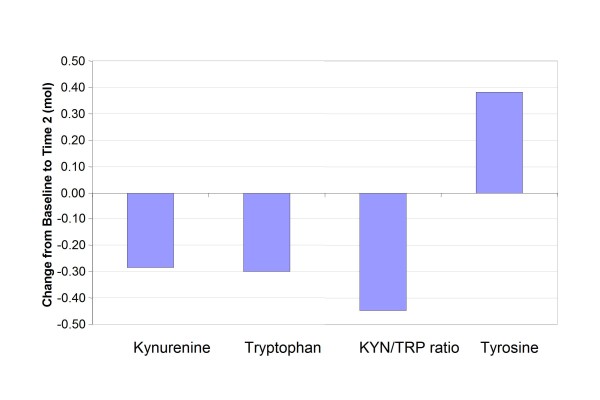
**Change in Biological Factors from Time 1 to Time 2 for Women with Breast Cancer**.

Variabilities in KYN and KYN/TRP ratios were much higher in the women with BCA than in women without BCA (comparison group) and noticeably higher levels of TYR were found for women with BCA (Figure 1). Women with BCA had a significantly higher KYN/TRP ratio (*p *= 0.036) than the women in the comparison group. There were no statistically significant differences between these women with regard to KYN (*p = *-0.18), TRP (*p *= 0.16), or TYR (*p *= 0.54). Means by measurement time-point for the women with BCA are plotted in Figure 2 and listed in Table 2. Although there were decreases in KYN, TRP, KYN/TRP ratio and increases in TYR in women with BCA from baseline to T2, these changes were not statistically significant.

**Table 2:** Mean Changes in Biological Factors in Women with Breast Cancer from Baseline (Time 1) to After Completion of Chemotherapy (Time 2)

	mean	median	std	stderr	min	max	**95% LCL**^**1**^	**95% UCL**^**2**^	p-values
Kynurenine	-0.28	0.08	1.12	0.21	-4.11	0.82	-0.70	0.13	0.91
Tryptophan	-0.30	-2.56	11.16	2.04	-17.12	25.68	-4.47	3.87	0.88
Kyn/Trp ratio	-0.45	0.25	2.00	0.37	-5.76	2.04	-1.20	0.30	0.92
Tyrosine	0.38	3.08	21.48	3.92	-31.55	53.50	-7.64	8.40	0.92

1 Upper control limit

2 Lower control limit

## Discussion

This pilot study was conducted to compare plasma concentrations of TRP, KYN, and TYR between women with BCA and women without cancer, and to determine how levels change over the course of BCA treatment. The sample of women with BCA in this study had lower levels of TRP and higher levels of KYN and TYR compared to women without BCA (comparison group). Although these differences did not reach statistical significance, the findings suggest that there may be quantitative differences in the levels of KYN and TYR. Because KYN and TYR are potentially neurotoxic, this finding needs to be further examined. Women with BCA also had a significantly higher KYN/TRP ratio compared to women without cancer. Findings of an elevated KYN/TRP ratio in this pilot study are consistent with one previous research study that found significantly higher IDO levels in serum and higher expression of IDO from breast tissue samples in 30 women with BCA compared to a control (no cancer) group [33]. In that study, the serum levels and expression of IDO did not correlate with histologic classification, tumor size, lymphatic or venous invasion, or lymph node metastasis but was significantly correlated with the serum level of immunosuppressive acidic protein (IAP), a biomarker of systemic immunodepression, suggesting that increased levels of IDO were induced by changes in immune function as opposed to breast tumor IDO secretion. Levels of TRP, KYN, and TYR were not reported, thus, no inferences were made about the effect of IDO on TRP degradation. However, the present study findings suggest that increased TRP degradation is present in women with BCA due to increased KYN along with decreased TRP.

The results of the current study support the need for further research of increased TRP degradation as a potential biological mechanism in breast cancer outcomes and symptom research. A deeper understanding of the relationships among neuropsychiatric symptoms and TRP degradation over the treatment trajectory may assist in the development of new symptom management approaches. For instance, IDO activity (measured indirectly by the KYN/TRP ratio) and an increased neurotoxic potential predict the occurrence of depression in patients with hepatitis C being treated with IFN-α based immunotherapy [34,35]. The neurotoxic potential (KYN/kynurenic acid ratio) is an indicator of the portion of KYN available to the N-methyl-D-aspartate (NMDA) receptor antagonist pathway and more precisely gauges the TRP and KYN levels within the central nervous system [36,37].

Consistent with previous research, the women with BCA had lower levels KYN and KYN/TRP ratio after the completion of chemotherapy [33], however the level of TRP continued to decline. While we cannot make any inferences about the effectiveness of chemotherapy in this study, past research findings suggest that the KYN/TRP ratio may be a useful indicator of treatment efficacy. Sakurai et al. [38] measured the KYN/TRP ratio in women with recurrent BCA who were receiving chemotherapy and compared levels between two different regimens, paclitaxel and docetaxel therapy. They found that women treated with docetaxel had higher KYN/TRP ratios post-chemotherapy compared to women receiving paclitaxel, suggesting the paxitaxel regimen may be more effective in eradicating IDO-secreting tumor cells. The KYN/TRP ratio also been used to predict prognosis and recurrence in patients with colorectal, lung, and ovarian cancer [26-29]. Given the multifactorial role of IDO and TRP, further research is necessary to determine the relationships among these important biological factors and neuropsychiatric symptoms, treatment efficacy, prognosis, and recurrence in women with BCA.

### Study limitations

There are several limitations of the present findings which should be discussed, most notably the small sample size in each group. Although the research team calculated the sample size estimate based on past research, a more robust sample size may have improved the power to detect significant differences in levels of TRP, KYN and TYR. In addition, findings of the study could also be critiqued in relation to the sample of women with BCA, which was primarily composed of women with early stage BCA and did not permit subgroup analysis by BCA stage. Systemic inflammatory processes play a key role in the initiation and progression of cancers and have been shown to potently induce IDO activity [39]. Finally, other biological factors that were not measured in this study, particularly, inflammatory molecules, can alter the activity of IDO both indirectly through the siphoning of TRP along the KYN pathway and by direct enhancement of their metabolism [14]. Conversely, drugs that inhibit IDO or TDO can potentially modify the levels of all these compounds, both directly and through changes in the availability of TRP [40]. Future research studies should consider measuring inflammatory markers and controlling for the type of chemotherapeutic regimen as a strategy to clarify how these factors influence TRP degradation and the development of neuropsychiatric symptoms in women with BCA.

## Conclusion

The sample of women with BCA in this study had lower mean levels of TRP, higher levels of KYN, and a significantly elevated KYN/TRP ratio, compared to women without BCA. In women with BCA, the KYN/TRP ratio remained elevated with reduced levels of TRP over the treatment trajectory. These findings are consistent with increased TRP degradation and support the need for further lines of inquiry about the relationships among TRP and TRP metabolites, other biological factors along the IDO-KYN pathway, and neuropsychiatric symptoms in women with BCA. Interrelationships among several key biological processes are likely to influence co-occuring symptoms associated with BCA and its treatment, including immune activation, stimulation of IDO activity, and subsequent TRP degradation. Systemic manifestations of TRP degradation may depend on the stage of BCA as well as the temporal nature of the disease process and degree of systemic inflammation.

## Competing interests

The authors declare that they have no competing interests.

## Authors' contributions

DEL conceived of the study and participated in its design and coordination, and helped to draft the manuscript. JMW participated in data collection, analysis and helped to draft the manuscript. ARS participated in data analysis and helped to draft the manuscript. CMS participated in the design of the study and performed the statistical analysis. NLM participated in the design of the study, data analysis and helped to draft the manuscript. All authors read and approved the final manuscript.
